# SIV Infection Facilitates *Mycobacterium tuberculosis* Infection of Rhesus Macaques

**DOI:** 10.3389/fmicb.2016.02174

**Published:** 2017-01-13

**Authors:** Ming Guo, Qiao-Yang Xian, Yan Rao, Jing Zhang, Yong Wang, Zhi-Xiang Huang, Xin Wang, Rong Bao, Li Zhou, Jin-Biao Liu, Zhi-Jiao Tang, De-yin Guo, Chuan Qin, Jie-Liang Li, Wen-Zhe Ho

**Affiliations:** ^1^School of Basic Medical Sciences, Center for Animal Experiment/Animal Biosafety Level III Laboratory, Wuhan UniversityWuhan, Hubei, China; ^2^Key Laboratory of Human Diseases Comparative Medicine, Ministry of Health, Institute of Laboratory Animal Science, Chinese Academy of Medical Sciences and Comparative Medicine Center, Peking Union Medical CollegeBeijing, China; ^3^Department of Pathology and Laboratory Medicine, Temple University Lewis Katz School of MedicinePhiladelphia, PA, USA

**Keywords:** SIV, HIV, *Mycobacterium tuberculosis*, coinfection, rhesus monkey

## Abstract

Tuberculosis (TB) is a common opportunistic infection and the leading cause of death for human immunodeficiency virus (HIV)-infected patients. Thus, it is necessary to understand the pathogenetic interactions between *M.tb* and HIV infection. In this study, we examined *M.tb* and/or simian immunodeficiency virus (SIV) infection of Chinese rhesus macaques. While there was little evidence that *M.tb* enhanced SIV infection of macaques, SIV could facilitate *M.tb* infection as demonstrated by X-rays, pathological and microbiological findings. Chest X-rays showed that co-infected animals had disseminated lesions in both left and right lungs, while *M.tb* mono-infected animals displayed the lesions only in right lungs. Necropsy of co-infected animals revealed a disseminated *M.tb* infection not only in the lungs but also in the extrapulmonary organs including spleen, pancreas, liver, kidney, and heart. The bacterial counts in the lungs, the bronchial lymph nodes, and the extrapulmonary organs of co-infected animals were significantly higher than those of *M.tb* mono-infected animals. The mechanistic studies demonstrated that two of three co-infected animals had lower levels of *M.tb* specific IFN-γ and IL-22 in PBMCs than *M.tb* mono-infected animals. These findings suggest that Chinese rhesus macaque is a suitable and alternative non-human primate model for SIV/*M.tb* coinfection studies. The impairment of the specific anti-TB immunity is likely to be a contributor of SIV-mediated enhancement *M.tb* infection.

## Introduction

*Mycobacterium tuberculosis* (*M.tb*) and human immunodeficiency virus (HIV) remain severe dual-epidemics globally. In 2014, an estimated 9.6 million people worldwide suffered from active tuberculosis (TB) and more than 34 million people were living with HIV (WHO, 2015). *M.tb* and HIV have been closely linked since the emergence of acquired immune deficiency syndrome (AIDS). Among the 9.6 million new TB infections worldwide, 1.2 million (13%) were HIV seropositive. In addition, HIV-infected individuals who are also infected with *M.tb* are much more likely to develop severe TB disease than those who are HIV negative. Of the 1.5 million died from TB in 2014, 0.4 million were co-infected with HIV (~27%) (WHO, 2015). Although HIV-related TB is both treatable and preventable, incidence continues to climb in developing countries where resources are limited. In addition, clinical data suggested that coinfection with HIV and *M.tb* increases progression of both diseases. Thus, it is of importance to understand the pathogenic interactions between *M.tb* and HIV.

The lack of insight about the interplays between HIV and *M.tb* is partially due to the absence of effective experimental animal models and difficulties in human studies (Mehra et al., 2011). Although small animals such as mice can be infected by *M.tb*, they are not the hosts of HIV. In contrast, nonhuman primates (NHP), such as Macaca (macaques: rhesus, cynomolgus), are susceptible to infection by simian immunodeficiency virus (SIV), a retrovirus similar to HIV, and can develop AIDS (Zhou et al., 2013). The SIV macaque models have been extensively used for HIV/AIDS research (Zhou et al., 2013). As a mainstay of NHP model of HIV/SIV research, Indian rhesus macaques have contributed greatly to our understanding of HIV disease. Indian rhesus macaques have been used to study the impact of SIV infection on *M.tb* infection (Safi et al., 2003; Mehra et al., 2011). However, the Indian rhesus macaque model has its limitations. For example, SIV-infected Indian rhesus macaques develop AIDS shortly after infection. In contrast, SIV-infected macaques of Chinese origin survived longer than Indian rhesus macaques (Ling et al., 2002; Burdo et al., 2005; Cumont et al., 2008; Zhou et al., 2013).

Macaques are also a good model for TB studies, as they can be infected by *M.tb*, progressing to TB disease that closely resembles the various manifestations of human TB (Capuano et al., 2003; Peña and Ho, 2015). Similar to human *M.tb* infection, infected macaques can develop acute TB or maintain TB latency for years (Gormus et al., 2004). Therefore, macaques are suitable for TB/HIV coinfection studies. There have been several studies using the macaque models (rhesus, pigtail or cynomolgus species) for *M.tb*/SIV coinfection studies (Shen et al., 2001, 2002; Diedrich et al., 2010; Mehra et al., 2011). These studies demonstrated that latently infected macaques with high-dose BCG (Shen et al., 2001, 2002) or low-dose Erdman or CD1551 strains (Diedrich et al., 2010; Mehra et al., 2011) could have TB reactivation when co-infected with SIV. While these findings are important in mimicking one aspect of HIV/*M.tb* coinfection, e.g., *M.tb* infection first and then SIV infection, there is still need to examine whether SIV infection facilitates *M.tb* infection and development of severe TB disease. In the present study, we used SIV-infected Chinese rhesus macaques to study the impact of SIV on *M.tb* infection.

## Materials and methods

### Ethics statement

All animals in this study were housed at the Animal Bio-Safety Level III (ABSL-III) Laboratory of Wuhan University School of Medicine. All housing and animal care procedures were in compliance with the China laws on animal experiments (Laboratory animal–Requirements of environment an housing facilities GB14925-2010, China; Regulations on administration of laboratory animals, Ministry of Science and Technology, 1988, China) and with the 8th Guide for the Care and Use of Laboratory Animals of Association (Committee for the Update of the Guide for the Care and Use of Laboratory Animals, 2011). The ABSL-III Laboratory is certified by the Association for Assessment and Accreditation of Laboratory Animal Care (AAALAC) International. All study protocols and procedures were approved by the Institutional Animal Care and Use Committee (IACUC) of Wuhan University School of Medicine prior to the experiments. All experimental procedures were performed under anesthesia with intramuscular injection of ketamine hydrochloride (10 mg/kg) and atropine (0.04 mg/kg), and all efforts were made to minimize suffering of the study animals. We followed the guidelines for humane euthanasia of rhesus macaques, and the re-defined humane endpoints including marked lethargy, severe dyspnea at rest, hemoptysis and loss of 25% body weight in 3 consecutive weeks compared to the body weight prior to *M.tb* infection. If any one of these conditions was present in the study animals, a humane endpoint was determined and the animals were euthanized within 24 h.

### Experimental animals

Nine Chinese origin female rhesus macaques (*M. mulatta*) of 5–6 years old were used for this study. The animals were purchased from Sichun Yibin Non-Human Primates Breeding and Research Center, Sichuan, China. These animals were strictly screened by an independent testing laboratory VRL Animal Health Services (AHS) to ensure that they were free of simian retrovirus D, SIV, simian T leukemia virus type 1 (STLV-1) and herpes virus B. In addition, the animals were quarantined for 90 days and tested by tuberculin skin test (TST) to ensure that they were free of *M.tb* prior to infection. The animals were individually housed in stainless steel wire-bottomed cages (80 × 80 × 85 cm dimension) with sufficient space supplied with a commercial monkey diet and water and twice daily with fresh fruit in an air-conditioned room and monitored by a computer-based system. After SIV or *M.tb* inoculation, all the study animals were transferred to the negative pressure rooms and individually housed in the negative pressure cages (isolators). These measurement ensured that the animals were not infecting each other.

### SIV and/or *M.tb* infection

The experimental designs of SIV and/or *M.tb* infection are described in Table 1. SIVmac239 strain was obtained from AIDS Research & Reference Reagent Program (Contributor: Dr. Ronald Desrosiers) of the National Institutes of Health (Bethesda, MD). *M.tb* H37Rv strain was obtained from the Research Institute of Tuberculosis (Tokyo, Japan). Six animals were intravenously inoculated with 0.5 mL of SIVmac239 (1.5 × 10^3^ TCID50) (Bao et al., 2014). Six weeks after SIV infection, three SIV mono-infected animals and three SIV uninfected animals were inoculated with *M.tb* H37Rv at the dose of 34 colony forming unit (CFU) by bronchoscopic instillation as described (Zhang et al., 2011). The plasma SIV RNAs were quantified by real-time PCR with the primers of SIV GAG gene as described (Bao et al., 2014; Liu et al., 2016).

**Table 1:** **Experimental design for SIV and/or *M.tb* infection**.

**Group**	**Animal ID**	**Gender**	**Age (Year)**	**Weight (kg)**	SIVmac239 (i.v.^*^) At Week 0	H37Rv (i.t.^*^) At Week 6	**Week of Necropsy (After *M.tb* infection)**
SIV + *M.tb*	WSP1	Female	5.4	5.86	+	+	16
	WSP2	Female	5.0	5.07	+	+	19
	WSP3	Female	6.1	6.00	+	+	18
SIV	WSP4	Female	5.0	5.36	+	-	NA
	WSP5	Female	5.3	5.89	+	-	NA
	WSP6	Female	5.1	5.03	+	-	NA
*M.tb*	WSP7	Female	6.3	5.15	-	+	NA
	WSP8	Female	5.0	5.20	-	+	25
	WSP9	Female	6.0	5.15	-	+	22

* *i.v., intravenous injection*;

* *i.t., intratracheal instillation*;

*NA, not available. SIVmac239 (1.5 × 10^3^ TCID50) in PBS (1 mL) was intravenously introduced into six animals at week 0. Six weeks later, three of the six SIV-infected animals and three uninfected animals were inoculated with M.tb H37Rv (34 CFU) by intratracheal instillation*.

### Clinical evaluation and chest X-rays

All the study animals were observed daily for appetite and every 2 or 3 weeks for body temperature and weight based on the score system described previously (Zhang et al., 2014). Appetite was scored daily by the amount of food taken per meal (2 = all; 1 = about half; 0 = none). A maximum appetite scores that an animal could achieve were 6 points per day and 42 points per week, which was defined as 100%. Blood samples collected from the animals were used for CRP (C-reactive protein), ESR (Erythrocyte sedimentation rate), flow cytometry and immunologic assays. ESR was measured by LENA (Linear chemicals, SL) system, CRP was determined by Biochemistry Analyzer (Hitachi 7080, Hitachi, Japan) with the reagents from Wako Pure Chemical Industries (Osaka, Japan). Chest X-rays were performed by a digital X-ray unit (SIEMENS Multimobil 2.5, Mobile 810 DDR system, and PiViewSTAR Workstation System). Evaluation of X-ray findings was performed by two radiologists with double-blind peer review.

### Flow cytometry analyses of CD4^+^ and CD8^+^ T cells

The CD4/CD8 ratios in peripheral blood were evaluated by flow cytometry using the fluorochrome labeled monoclonal antibodies: CD3-FITC (clone SP34; BD Pharmingen); CD4-PerCP (clone L200; BD Pharmingen); and CD8-PE (clone RPA-T8; BD Pharmingen). Antibodies, diluted as per manufacturer's instructions, were added to 100 μL of EDTA-whole blood and incubated for 30 min at room temperature. Erythrocytes were then lysed by BD FACS lysing solution (Becton, Dickinson). Cells were washed once with 1 × PBS and resuspended in 1 × PBS containing 1% paraformaldehyde. Unstained and single color control samples were acquired to calculate the compensation matrix. Gating was performed on mononuclear cells and then on CD3^+^CD4^+^ or CD3^+^CD8^+^ subpopulations. The flow cytometry analysis was performed on a BD FACSCalibur (Becton Dickinson Biosciences, San Jose, CA) and analyzed using CellQuest software (Becton Dickinson).

### ELISpot assay

96-well multiscreen-immunoprecipitation filtration plates (Millipore Corp., Bedford, MA) were hydrated with 70% ethanol, followed by washing with 1 × PBS three times, then coated with anti-human/macaque IFN-γ monoclonal antibody (U-Cytech Biosciences, Utrecht, The Netherlands) at 4°C for 24 h prior to the ELISpot assay (U-Cytech Biosciences). Blood from study animals was collected in heparin CPT Vacutainer tubes (Becton Dickinson Vacutainer Systems, Franklin Lakes, NJ) and centrifuged at 1750 × g for 30 min. The upper layer of peripheral blood mononuclear cells (PBMCs) was collected and washed twice with RPMI 1640 medium supplemented with 1% FBS (Gibco, Life Technologies). Freshly isolated PBMCs were resuspended at 1 × 10^6^ cells/mL in ELISpot medium (RPMI 1640 medium supplemented with 10% FBS) and added into the plates at 3 × 10^5^ cells/well. Cells were stimulated with PPD (10 μg/mL) from *M.tb* or concanavalin A (ConA, 5 μg/mL; Sigma, St. Louis, MO) or ELISpot medium only in duplicate wells for 24 h in 5% CO_2_ air at 37°C. PPD were prepared as described (Landi and Held, 1965; Brel et al., 1995). The numbers of spots were counted by an ELISPOT reader (Bioreader 4000, BIOSYS, Germany) and data are reported as the number of spot-forming units (SFUs) per million PBMCs.

### Real-time RT-PCR

The above isolated PBMCs were also treated with 10 μg/mL of PPD for 16 h at 37°C in a 5% CO_2_ incubator. After treatment, the cells were centrifuged at 400 × g for 5 min and the pellet was lysed in Tri-Reagent (Molecular Research Center Inc., Cincinnati, OH). Total cellular RNA was subjected to reverse transcription using the reagents from Promega (Madison, WI). The real-time PCR for the quantification of mRNA levels of IFN-γ and IL-22 was performed with the iQ SYBR Green Supermix (Bio-Rad Laboratories, Hercules, CA). The levels of glyceraldehyde-3-phosphate dehydrogenase (GAPDH) mRNA were used as an endogenous reference to normalize the quantities of cytokine mRNAs. For IFN-γ, primers are 5′-CCAAGTGATGGCTGAACTGTCG-3′ and 5′-TCTGACTCCTTTTTCGCTTCCC-3′. For IL-22, primers are 5′-TCCGCGGAGTCAGTATGAGTGAGC-3′ and 5′-GAACCTATCCGATTGAGGGAGCAGC-3′. GAPDH primers are 5′-GTCTGGAAAAACCTGCCAAG-3′ and 5′-ACCTGGTGCTCAGTGTAGCC-3′.

### Necropsy

Animals that reached the humane endpoints were euthanized first by anesthesia with ketamine (i.m. 10 mg/kg) and then by an intravenous overdose of sodium pentobarbital (100 mg/kg). A full necropsy was performed to obtain lungs and other organs under sterile conditions for microbiologic assays. A gross pathology scoring system (Lin et al., 2006; Marsollier et al., 2007) was used to quantitatively document TB lesions. The presence, number, size and distribution of granulomas in tissues of infected organs were documented.

### Bacterial burden

During the post-mortem, the tissues were randomly taken from different organs (lung, bronchial lymph node (BrLN), liver, spleen, kidney and pancreas) of the animals and examined for bacterial burden. Samples were homogenized in 5 mL PBS by a tissue homogenizer PRO250 (PRO Scientific, Monroe, CT). All samples were serially 10-fold diluted in PBS prior to being plated onto Middlebrook 7H11 medium (Difco, BD, USA) plus OADC supplements (Difco) and cycloheximid (100 μg/mL; Sigma, St. Louis, MO). After spreading the homogenate solution, plates were incubated at 37°C and counted for formed colonies 3–5 weeks after incubation.

### Statistical analysis

Data were analyzed using Prism6.0 (Graphpad Software, San Diego, CA) and SPSS software (version 16.0, SPSS, Chicago, IL, USA). Survival rates of the study animals were analyzed by the log-rank test. The significant differences in the clinical evaluations (appetite, body weight, body temperature, CRP and ESR), SIV RNA levels and CD4/CD8 T cells ratios between two groups were determined by the paired *t*-tests for area under the curve (AUC) of each animal. Student *t*-tests were performed on the differences in IFN-γ and IL-22 levels between two groups. The grosspathology and the bacterial burden between two groups were analyzed using Mann-Whitney test, *p* < 0.05 was considered statistically significant.

## Results

### SIV facilitates fatality of *M.tb*-infected macaques

**Figure 1:**
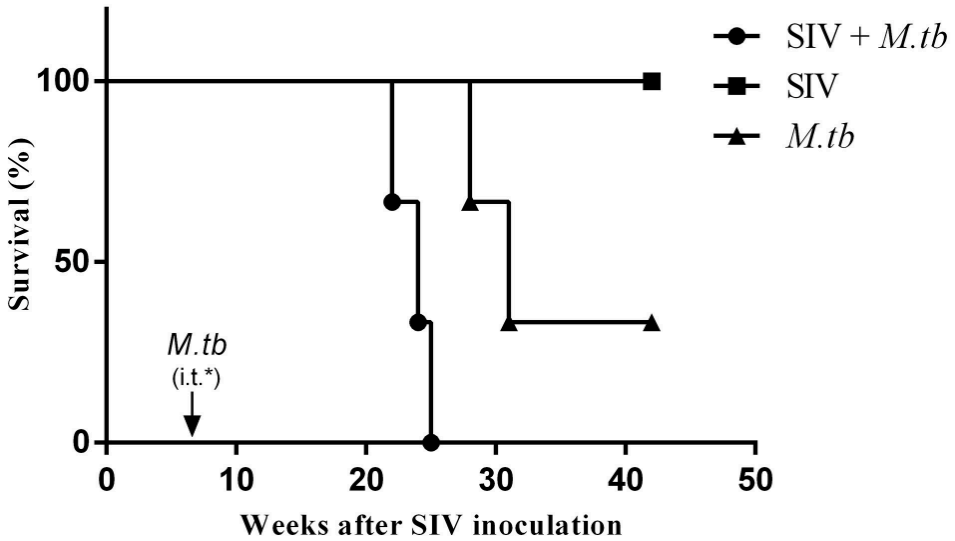
**Survival of animals in *M.tb* mono-infected and co-infected groups**. For the coinfection experiments, the animals were intravenously (i.v.) infected with SIVmac239 (TCID_50_ 1.5 × 10^3^) 6 weeks prior to inoculation by intratracheal instillation (i.t.) of *M.tb* H37Rv (34 CFU) (arrow). *M.tb* mono-infected animals were inoculated with the same dose of *M.tb* only. The survival rates were analyzed by the Log-rank test. The survival times of co-infected animals were significantly shorter (^*^P = 0.0246) than those of *M.tb* mono-infected animals.

All co-infected animals reached the humane endpoints with the survival time of 16 weeks (WSP1), 18 weeks (WSP3) and 19 weeks (WSP2) after *M.tb* infection, respectively (Table 1). In contrast, *M.tb* mono-infected animals had longer survival times than co-infected animals, as two of them survived 22 weeks (WSP9) and 25 (WSP8) weeks after *M.tb* infection, respectively (Table 1). The third one (WSP7) had the high levels of *M.tb* specific IFN-γ in PBMC (**Figure 5C**) and survived throughout the course (31 weeks) of the study. During the followed up (additional 24 months) this animal showed normal appetite, increased body weight and normal chest X-ray (data not shown). All SIV mono-infected animals did not develop AIDS or opportunistic infections and survived through the course of the study. Differences in survival times between co-infected and *M.tb* mono-infected animals were statistically significant (*P* = 0.0246) (Figure 1).

### Effects of SIV and/or *M.tb* infections on appetite, body weight, and body temperature

**Figure 2:**
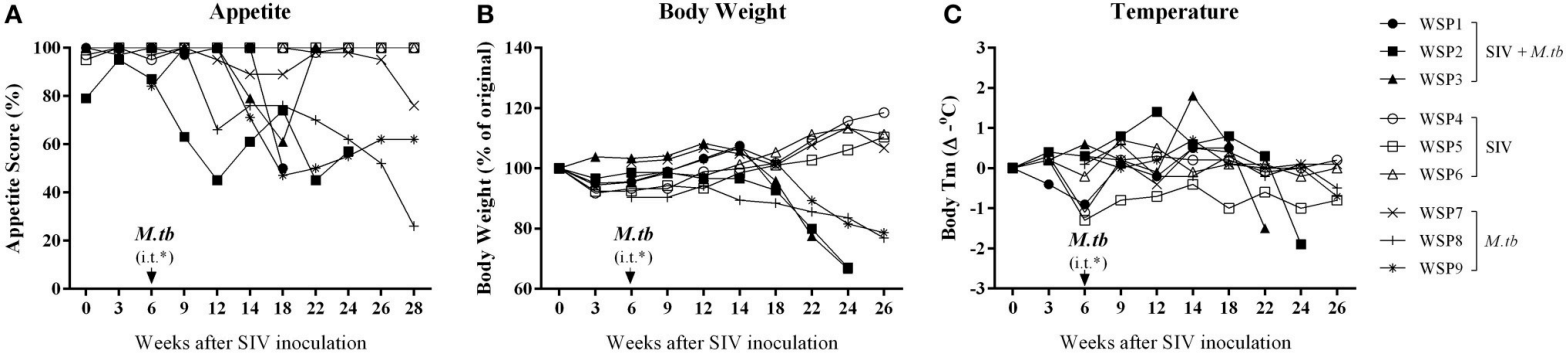
**Clinical manifestations. (A)** Weekly appetite score. Appetite was scored by the amount of food taken per meal (2 = all; 1 = about half; 0 = none). A maximum appetite scores that an animal could achieve was 6 points per day and 42 points per week, which was defined as 100%. A percentage of the appetite scores for each animal was calculated as weekly scores divided by 42. The appetite scores of co-infected animals were significantly lower (^*^P = 0.0212) than those of *M.tb* mono-infected animals 6 to 26 weeks after *M.tb* infection. **(B)** Percentage change in body weight (every 2 or 3 weeks). The weight loss of co-infected animals were significantly higher (^*^P = 0.0473) than that of *M.tb* mono-infected animals after *M.tb* infection (week 6 to week 26). **(C)** Changes in body temperature are measured as changes in °C (every 2 or 3 weeks). The body temperatures of co-infected animals were significantly higher (^**^P = 0.0052) than those of *M.tb* mono-infected animals 6 to 18 weeks after *M.tb* infection. Data are presented as individuals in each group. ^*^i.t., intratracheal instillation of *M.tb* (34 CFU).

Low grade fever, loss of appetite and weight are common symptoms of *M.tb* infection in humans. Thus, we examined these clinical parameters in the study animals. Following *M.tb* infection, all co-infected animals and two *M.tb* mono-infected animals (WSP8 and WSP9) exhibited loss of appetite and body weight (Figures 2A,B). One *M.tb* mono-infected animals (WSP7) exhibited only minor loss of appetite but little change in body weight. Co-infected animals exhibited significant loss of appetite and body weight than *M.tb* mono-infected group (*P* = 0.0212 and 0.0473, respectively). The animal WSP02 developed a fever at week 3 after *M.tb* infection and reached its highest level (1.4°C higher than the base level) at week 6 after *M.tb* infection. The animal WSP03 developed a fever with its maximum (1.8°C higher than the base level) at week 8 after *M.tb* infection. Two animals (WSP8 and WSP9) with mono-infection had low grade fever (0.6 and 0.7°C higher) at week 3 and week 6 (Figure 2C). Two (WSP2 and WSP3) co-infected animals had a significant drop of body temperature at the late stage of *M.tb* infection (Figure 2C). Co-infected animals had significantly higher body temperatures at the early and middle stage of *M.tb* infection (week 6 to week 18) than *M.tb* mono-infected animals (*P* = 0.0052). In contrast, SIV mono-infected animals had little changes of appetite, body weight and body temperature (Figure 2).

### *M.tb* mono-infection or *M.tb*/SIV coinfection increases ESR and CRP

**Figure 3:**
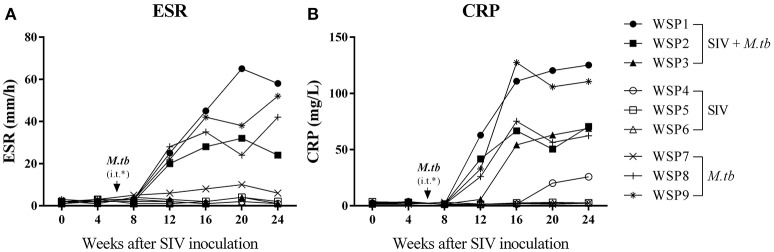
**ESR and CRP levels**. Peripheral blood was collected from the study animals at the indicated time points. The levels of Erythrocyte sedimentation rate (ESR) **(A)**, plasma levels of C-reactive protein (CRP) **(B)** were measured. ^*^i.t., intratracheal instillation of *M.tb* (34 CFU).

ESR and CRP are two inflammatory markers in active *M.tb* infection. As shown in Figure 3, two co-infected animals (WSP1 and WSP2) and all *M.tb* mono-infected animals showed elevated levels of ESR during the course of *M.tb* infection. All co-infected animals and two *M.tb* mono-infected animals (WSP8 and WSP9) demonstrated elevated levels of CRP starting at week 12 after SIV infection (Figure 3). WSP3 had normal ESR levels but abnormal levels of CRP (Figure 3B). The levels of ESR and CRP were not significantly different between co-infected and *M.tb* mono-infected animals (*P* = 0.9861 and 0.5963, respectively) (Figure 3). SIV mono-infected animals did not show elevated levels of ESR and CRP (Figure 3).

### *M.tb* infection has little impact on plasma SIV RNA and CD4/CD8 ratio

**Figure 4:**
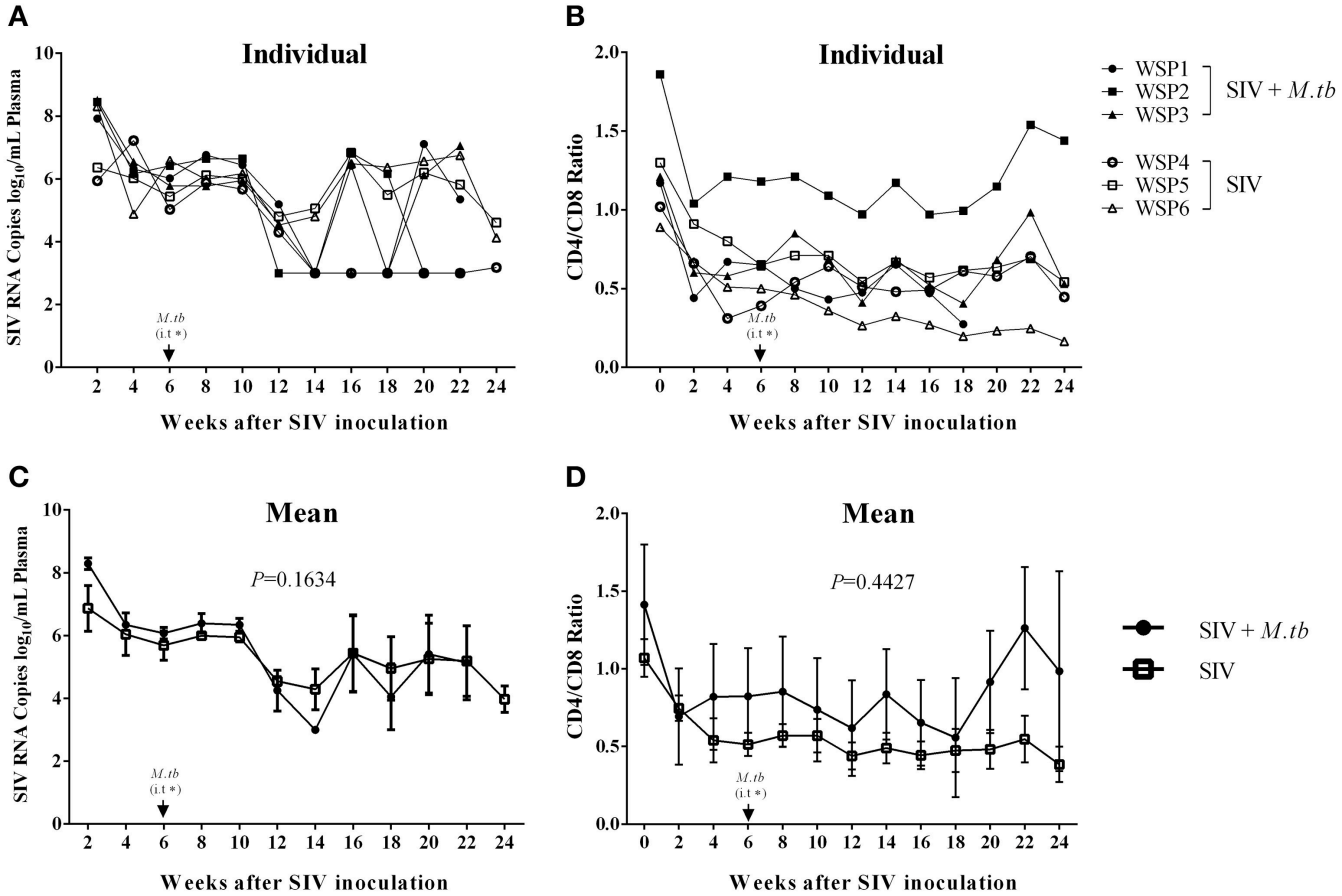
**Plasma SIV RNA levels and CD4/CD8 T cell ratios in PBMCs. (A)** Plasma SIV RNA copies were determined by the real time PCR with the primers of SIV GAG gene. The data shown are the means of plasma SIV RNA copies/mL of animals in two groups. **(B)** Flow cytometry analysis of CD4^+^ and CD8^+^ T cells in the peripheral blood. The data shown are the means of CD4/CD8 T cell ratios in PBMCs of three animals in two groups. **(C,D)** The means of SIV RNA levels **(C)** or CD4/CD8 ratios **(D)** in each group. The *P*-values of the parameters are indicated. ^*^i.t., intratracheal instillation of *M.tb* (34 CFU).

To determine the impact of *M.tb* on SIV infection, we measured the plasma SIV RNA levels of co-infected animals and SIV mono-infected animals. Plasma SIV RNAs were detected in all co-infected and SIV mono-infected animals 2 weeks after SIV infection. However, there was no significant difference (*P* = 0.1634) in viral RNA copies between co-infected and SIV mono-infected animals during the course of infection (Figure 4A). In addition, decreased CD4/CD8 ratios were observed in both co-infected and SIV mono-infected animals (Figure 4B), but there was little difference (*P* = 0.4427) between the two groups (Figure 4B).

### Effect of SIV infection on *M.tb* specific IFN-γ and IL-22

**Figure 5:**
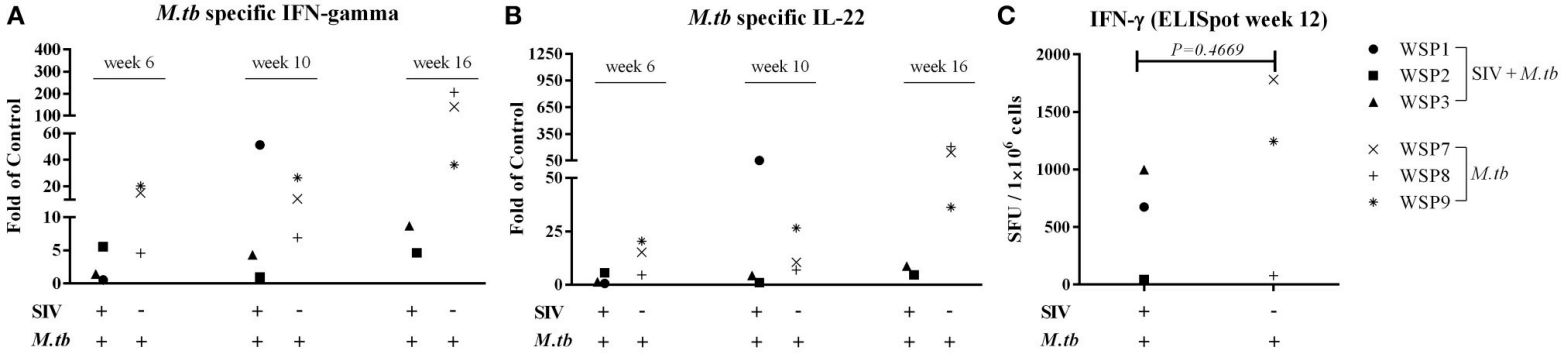
**Effect of *M.tb* and/or SIV infections on *M.tb* specific IFN-γ and IL-22**. PBMCs from the study animals were cultured in the presence of PPD, (10 μg/mL) for 16 h. Total RNAs were extracted and the expression of IFN-γ **(A)** and IL-22 **(B)** was measured by the real-time PCR at different time points (6 week, 10 week and 16 week after *M.tb* inoculation). The levels of IFN-γ and IL-22 mRNAs were expressed as fold changes of medium alone (control value = 1). The levels of IFN-γ and IL-22 mRNAs were not significantly different between co-infected and *M.tb* mono-infected animals (*P* > 0.05). IFN-γ ELISpot assay was performed in the presence of PPD as described in Materials and Methods. **(C)** The graphic representation of quantified numbers of PPD-induced IFN-γ spots are shown as SFU/1 × 10^6^ cells. The number of PPD-induced IFN-γ spots were not significantly different between co-infected and *M.tb* mono-infected animals (*P* = 0.4669).

IFN-γ is crucial in the host immunity against *M.tb* infection, as it restricts mycobacterial proliferation in macrophages and enhances CD8^+^ T cell-mediated CTL activity against *M.tb*-infected target cells (Flynn et al., 1993; Cooper and Khader, 2008). In addition to IFN-γ, IL-22 can also inhibit intracellular *M.tb* growth in macrophages by enhancing phagolysosomal fusion (Dhiman et al., 2009). Thus, we evaluated the expression of these cytokines in PBMCs of *M.tb*-infected animals. PPD-stimulated PBMCs from co-infected animals and *M.tb* mono-infected animals expressed IFN-γ (Figure 5A) and IL-22 (Figure 5B). However, the levels of PPD-induced IFN-γ and IL-22 mRNAs were lower in two of co-infected animals than those in *M.tb* mono-infected animals. The ELISpot assay also showed that two co-infected animals had lower frequency of PPD specific IFN-γ-secreting cells than *M.tb* mono-infected animals (Figure 5C). However, because one (WSP1) of co-infected animals had high levels of *M.tb*-induced IFN-γ and IL-22, differences in the levels of these cytokines between two groups were not significant (Figure 5). All SIV mono-infected animals lacked the PPD-stimulated IFN-γ and/or IL-22 responses at both mRNA and protein levels (Figure 5).

### SIV enhances *M.tb* infection of lungs and extrapulmonary organs

**Figure 6:**
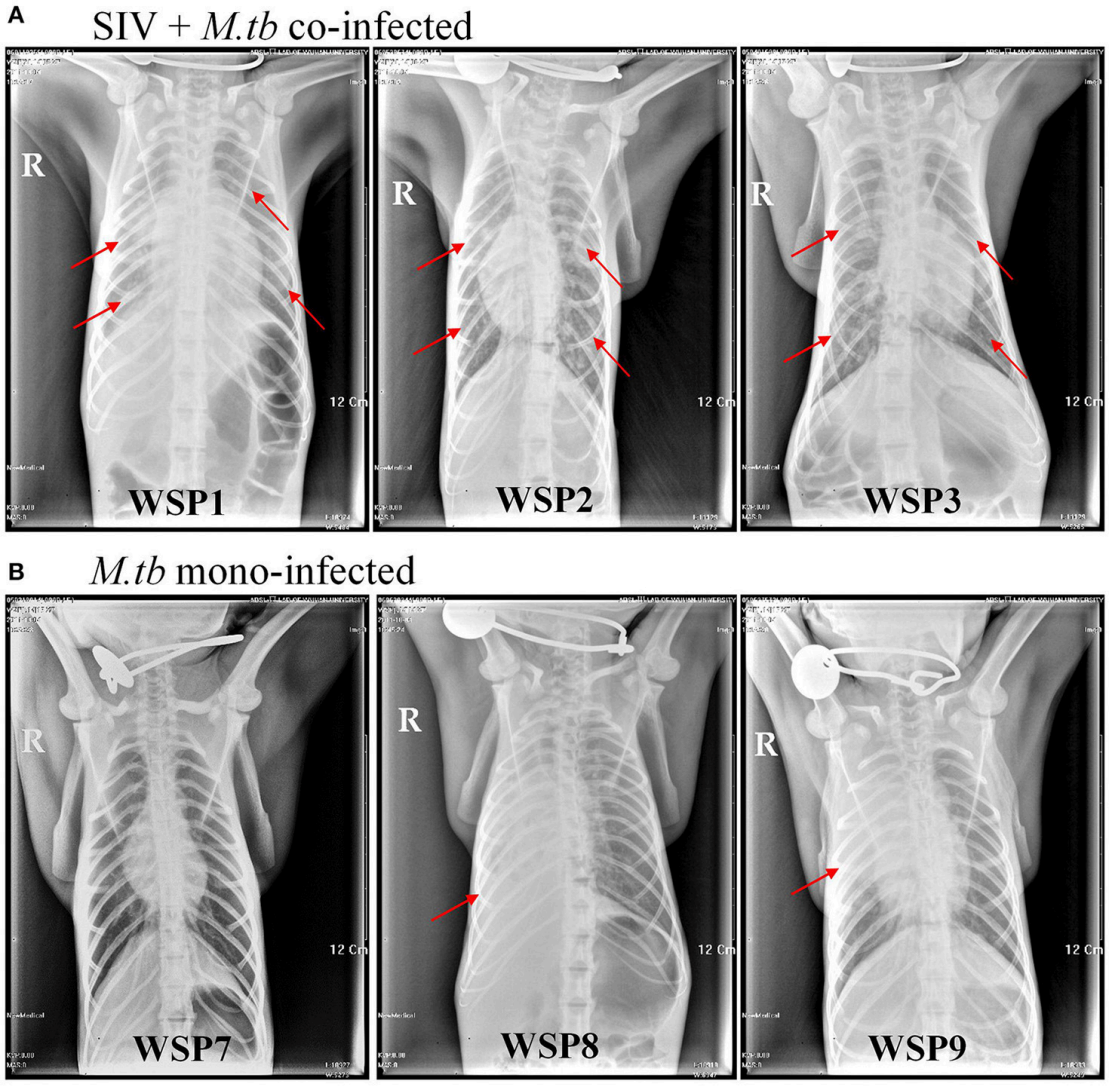
**Chest X-rays of co-infected and *M.tb* mono-infected animals**. Chest radiographs of co-infected and *M.tb* mono-infected animals at 14 weeks after *M.tb* infection. The red arrows indicate the positions of patchy nodular areas in lungs.

**Figure 7:**
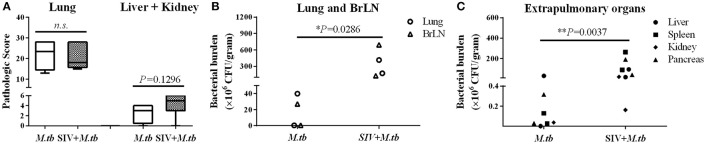
**Gross pathologic evaluation of TB granulomas and bacterial burdens in lungs and extrapulmonary organs of *M.tb* mono-infected and co-infected animals. (A)** The pathological scores were calculated based on the size and frequency of TB granulomas in the lungs, livers, spleens and kidneys. **(B)** The bacterial burdens in the lung and bronchial lymph node (BrLN) of co-infected animals are significantly higher (^*^P = 0.0286) than those of *M.tb* mono-infected animals. **(C)** The bacterial burdens in the extrapulmonary organs (liver, spleen, kidney, and pancreas) of co-infected animals were significantly higher (^**^P = 0.0037) than those of *M.tb* mono-infected animals.

**Figure 8:**
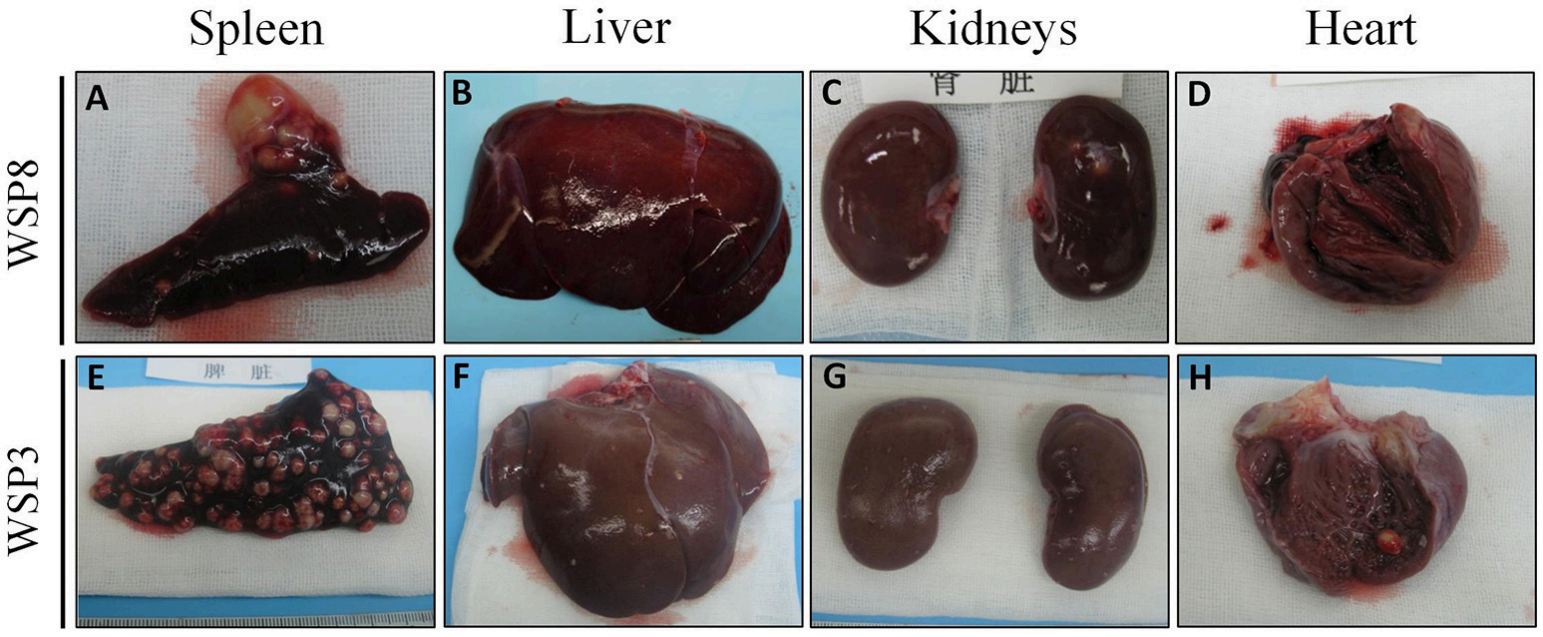
**Gross morphological pathology of extrapulmonary organs of the study animals**. A few granulomas were observed in spleen **(A)** and kidneys **(C)**, while no visible lesions in liver **(B)** and heart **(D)** of *M.tb* mono-infected animal (WSP8) at necropsy. A number of granulomas were observed in the spleen **(E)**, liver **(F)**, kidneys **(G)**, and heart **(H)** of co-infected animal (WSP3) at necropsy.

Eight weeks after *M.tb* infection, two (WSP8 and WSP9) of *M.tb* mono-infected animals showed abnormal chest X-rays. WSP8 had an area of consolidation in the right lower lobe, and WSP9 displayed an area of consolidation in the right middle lobe. In contrast, all co-infected animals showed multiple pulmonary nodules disseminated in the lungs. Fourteen weeks after *M.tb* infection, among *M.tb* mono-infected animals, posteroanterior chest X-rays of WSP8 showed areas of consolidation in the right upper lobe and right middle lobe (Figure 6B). WSP9 showed consolidation of upper and middle lobes. However, WSP7 remained a normal chest X-rays (Figure 6B). In contrast, all co-infected animals had multiple patches and nodular infiltrates distributed in both right and left lobes of lungs with miliary (Figure 6A). In addition, necropsy of co-infected animals revealed disseminated *M.tb* infections not only in the lungs but also in the extrapulmonary organs, including spleen, pancreas, liver, kidney and heart. The numbers of visible nodules in the extrapulmonary organs (kidneys and livers) of co-infected animals were higher than those of *M.tb* mono-infected animals (Figures 7A, 8). In addition, the bacterial counts in the lungs, BrLN and extrapulmonary organs (liver, kidney, heart, spleen) of co-infected animals were significantly higher than those of *M.tb* mono-infected animals (*P* = 0.0286 and 0.0037, respectively) (Figures 7B,C, and Supplementary Table 1). Except for one *M.tb* mono-infected animal (WSP7), all other five animals showed histopathologic evidence of extensive *M.tb* infection and caseous necrosis in the lungs. SIV/*M.tb* co-infected animals exhibited disseminated and more severe *M.tb* infection (appearance of granulomas) in multiple organs than *M.tb* mono-infected animals (Figure 8).

## Discussion

TB is one of the most common opportunistic infections for people infected with HIV, even when CD4 cells count and antiretroviral therapy are taken into account (Suchindran et al., 2009). Because people with HIV/AIDS carry a high risk of *M.tb* infection and disease severity, it is clinically significant to have an experimental animal model that mimics *M.tb* and HIV coinfection. However, unreliable and limited animal models restrict the research on HIV and *M.tb* coinfection. Macaques have been widely used for HIV/AIDS research as they can be infected by SIV (Zhou et al., 2013). In addition, cynomolgus and rhesus macaques are the host for *M.tb* infection (Walsh et al., 1996; McMurray, 2000; Verreck et al., 2009). Therefore, the macaques represent a suitable animal model for either SIV or *M.tb* infection. However, there are limited studies that used the macaque models to study SIV and *M.tb* coinfection. Those studies (Safi et al., 2003; Diedrich et al., 2010; Mehra et al., 2011) using cynomolgus or rhesus macaques of Indian origin demonstrated that rhesus macaque is a suitable model to study SIV and *M.tb* coinfection. It is unclear, however whether macaques of Chinese origin can be used for SIV and *M.tb* coinfection studies. Although Chinese macaques and Indian macaques belong to the same species, they demonstrate differences in physiological and immunological responses, as well as genetic background (Champoux et al., 1997; Clarke and O'Neil, 1999; Viray et al., 2001; Doxiadis et al., 2003; Penedo et al., 2005; Otting et al., 2007). Therefore, it is of importance and interest to examine the feasibility of using Chinese macaques for SIV and *M.tb* coinfection.

To mimic clinical situation where HIV-infected individuals are susceptible to *M.tb* infection and develop severe TB disease, we first infected the animals with SIV for 6 weeks prior to *M.tb* inoculation. Because Chinese rhesus macaques are highly susceptible to *M.tb* Erdman strain infection, developing acute TB disease regardless of the challenging doses (Zhang et al., 2014), we used *M.tb* H37Rv strain to infect the animals. We found that all co-infected and two out of three *M.tb* mono-infected animals developed the end-stage of disease. Compared to *M.tb* mono-infected animals, co-infected animals reached to the humane endpoint faster (Figure 1). At necropsy, we observed a number of granulomas in the lungs of co-infected and *M.tb* mono-infected animals. There were no significant differences in the pathologic scores of the lungs between the two groups (Figure 7A). This observation could be due to the fact that the animals in both groups developed acute/severe *M.tb* infection, resulting in extensive lung damages. However, chest X-rays showed that co-infected animals had disseminated *M.tb* infection in both right and left lungs (Figure 6), while *M.tb* mono-infected animals had the infection limited in their right lungs (Figure 6). In addition, co-infected animals had more granulomas in the extrapulmonary organs (kidneys and livers) than *M.tb* mono-infected animals (Figure 7A). Interestingly, a granuloma was found in the heart of a co-infected animal (WSP3) (Figure 8). The bacterial burdens in the lungs, BrLNs and extrapulmonary organs (liver, kidney, heart, spleen) of co-infected animals were significantly higher than those of *M.tb* mono-infected animals (Figures 7B,C; Supplementary Table 1). In addition, co-infected animals survived shorter time than *M.tb* mono-infected animals. These findings indicate that SIV infection results in the spread of *M.tb*, leading to disseminated *M.tb* infection in multiple organs of co-infected animals. Interestingly, our data (Figure 4) showed that *M.tb* infection had little impact on SIV infection, as there were little differences in the viral loads and CD4/CD8 ratios between co-infected and SIV mono-infected animals. Shen et al. showed that BCG coinfection enhanced the destruction of CD4^+^ T cells in SIV-infected macaques whose viral loads were high (Shen et al., 2002). This discrepancy could be due to the differences in *M.tb* strains, SIV strains and macaque species (Rhesus, Pigtailed vs. Chinese rhesus) used in the studies. Evidently, more studies with large numbers of macaques are needed in order to draw the conclusion.

Whilst HIV/SIV-mediated immunological impairments are likely to contribute to the increased morbidity and mortality associated with *M.tb* infection, it is unclear, however, HIV/SIV impair specific anti-TB immunity. To determine the specific mechanisms for SIV infection-mediated enhancement of *M.tb* infection, we examined two key cytokines (IFN-γ and IL-22) that have the ability to inhibit *M.tb* infection (Luciw et al., 2011; Mehra et al., 2011, 2012; Dutta et al., 2012). IFN-γ is well known to have a key role in the immune defenses against *M.tb* infection. The early study showed that persons with genetic defects and reduced production of IFN-γ or failure to respond to IFN-γ develop severe and fatal mycobacterial disease (Safi et al., 2003). IL-22-producing cells at mucosal surfaces play a crucial role in the host innate defense against microorganisms, including *M.tb* (Aujla and Kolls, 2009). A recent report showed that IL-22 can inhibit the growth of *M.tb* in macrophages by enhancing phagolysosomal fusion (Dhiman et al., 2014). In addition, the IL-22-producing T cell-mediated immunity is important for the protective anti-*M.tb* response (Bhuju et al., 2012). IL-22 is mainly produced by IFN-γ-secreting cells but is dispensable for host protection against *M.tb* infection (Behrends et al., 2013). There is a reciprocal relationship between IFN-γ and IL-22 production by IL-22 and IFN-γ-producing CD4^+^ T cells (Qiu et al., 2013). We found that the levels of the *M.tb* specific IL-22 were significantly lower in two of co-infected animals than those in *M.tb* mono-infected animals (Figure 5B). In addition, the productions of *M.tb* specific IFN-γ were also suppressed in two co-infected animals (Figure 5C). These findings, however, need to be confirmed in future studies with large number of animals.

In summary, we demonstrated that Chinese macaque is a suitable and alternative NHP model for SIV/*M.tb* coinfection studies. With limited number of animals, we observed that while *M.tb* had little impact on SIV infection, SIV infection accelerated *M.tb* dissemination in multiple organs, including heart in co-infected animals. The impairment of the specific anti-TB immunity is likely to be a key contributor of SIV-mediated *M.tb* dissemination. However, further investigations on interactions between HIV/SIV and *M.tb* are necessary, which should improve and advance our knowledge about treating HIV and *M.tb* co-infected individuals.

## Author contributions

Conceived and designed the study: MG, CQ, DG, and WH. Performed the study: MG, QX, YR, JZ, YW, ZH, XW, RB, LZ, J-BL, and ZT. Analyzed the data: MG and J-LL. Wrote the manuscript: MG, J-LL, and WH. All authors read, edited and approved the final manuscript.

## Funding

This work was supported by grants from The National Science and Technology Major Projects of Infectious Disease (2012ZX10004501-001-004, 2013ZX10003009-003, and 2014ZX10001003), the National Natural Sciences Foundation of China (81201261, 81301428, 81271334, and 81571962), and the Natural Science Foundation of Hubei (2014CFA076).

## Ethics statement

This project was approved by the IACUC of Wuhan University School of Medicine (A32011003).

### Conflict of interest statement

The authors declare that the research was conducted in the absence of any commercial or financial relationships that could be construed as a potential conflict of interest.
