# Supplementary material for: SIV Infection Facilitates Mycobacterium tuberculosis Infection of Rhesus Macaques
Source: Front Microbiol. 2017 Jan 13;7:2174. doi: 10.3389/fmicb.2016.02174 (PMC5233680; doi:10.3389/fmicb.2016.02174)
Supplement: Supplementary file 1 [file Table1.docx]

Supplementary Material

**SIV infection facilitates *Mycobacterium tuberculosis* infection of Rhesus Macaques**

**Ming Guo, Qiao-Yang Xian, Yan Rao, Jing Zhang, Yong Wang, Zhi-Xiang Huang, Xin Wang, Rong Bao, Li Zhou, Jin-Biao Liu, Zhi-Jiao Tang, De-yin Guo, Chuan Qin, Jie-Liang Li, and Wen-Zhe Ho^*^**

*** Correspondence:** Wenzhe-Ho: wenzheho@temple.edu

# Supplementary Tables

**Supplementary Table 1. Bacterial burden in *M.tb*-infected organs**

| **Group** | **Animal ID** | **CFU/gram** | | | | | |
| --- | --- | --- | --- | --- | --- | --- | --- |
|  |  | **Lung** | **BrLN*** | **Liver** | **Spleen** | **Kidney** | **Pancreas** |
| SIV+*M.tb*  Co-infected | WSP1** | N/E | N/E | N/E | N/E | N/E | N/E |
|  | WSP2 | 4.17×10^8^ | 1.29×10^8^ | 9.33×10^7^ | 8.91×10^7^ | 2.09×10^7^ | 4.07×10^7^ |
|  | WSP3 | 1.74×10^8^ | 6.92×10^8^ | 1.74×10^7^ | 2.63×10^8^ | 1.62×10^5^ | 1.91×10^8^ |
| *M.tb*  Mono-infected | WSP7*** | N/E | N/E | N/E | N/E | N/E | N/E |
|  | WSP8 | 3.98×10^7^ | 2.69×10^7^ | 2.95×10^4^ | 2.75×10^4^ | 3.72×10^4^ | 3.16×10^5^ |
|  | WSP9 | 4.07×10^5^ | 6.17×10^5^ | 7.24×10^2^ | 1.29×10^5^ | 0 | 2.95×10^4^ |

*BrLN: Bronchial lymph nodes

**N/E: This animal was not evaluated due to the contaminations of its specimens.

***N/E: This animal didn’t reach the humane endpoint at the end of the study.
